# Dissociative Identity Unveiled: A Case Report of 17 Distinct Identities Emerging in a Clear Timeline Following Trauma Events

**DOI:** 10.1192/j.eurpsy.2024.1715

**Published:** 2024-08-27

**Authors:** J. Li, N. M. Childs, H. Raai

**Affiliations:** ^1^Department of Psychiatry and Behavioral Health, SBH Health System; ^2^Medicine, CUNY School of Medicine, New York, United States

## Abstract

**Introduction:**

Dissociative Identity Disorder (DID) is a complex and enigmatic mental disorder in which an individual maintains two or more distinct identities or personality states. We present a rare and captivating case report of a 27-year-old female patient who exhibited a remarkable 17 distinct identities, developed in a clear and unprecedented timeline following a series of specific traumatic events. The novelty of this case lies in the comprehensive documentation and analysis of the sequential emergence of these identities, offering valuable insights into the development and progression of DID.

**Objectives:**

Our aim in presenting this case study is to offer a unique presentation to the constantly evolving understanding of DID. This case offers insight and provokes the need for research into the traumagenic nature of DID. This case showcases the influence on the chronological evolution of the patient’s 17 identities following a multitude of traumatic events.

**Methods:**

Structured interviews, psychiatric assessments, and psychological measurements, including self-reported measures of the Dissociative Experiences Scale, were employed to assess the identities and their individual experiences of the traumatic events. The patient was diagnosed with DID and received treatment including pharmacotherapy, psychoeducation, and trauma-focused psychotherapy. As a result of the therapeutic process, the patient was able to develop a higher sense of self-awareness and thus was able to integrate their 17 fragmented identities into a single host identity, demonstrating improvement in the functioning of interpersonal relationships.

**Results:**

The patient’s history reveals that the onset of her DID was linked to a traumatic event that occurred during early childhood, triggering the emergence of her first alternate identity. Over time, additional identities manifested, each appearing to serve as a coping mechanism to contend with the psychological distress stemming from subsequent significant trauma episodes. This case report meticulously outlines the chronological development of these identities and explores the distinct characteristics, behaviors, and roles assumed by each personality.

**Conclusions:**

This case report offers valuable information on the complex development and pathogenesis of DID with a trauma influence. The presentation of this patient may lead to further research and more tailored therapeutic interventions for individuals suffering from DID.

**Disclosure of Interest:**

None Declared

